# Retraction: Structural characterization of ginseng cyclopeptides and detection of capability to induce apoptosis in gastrointestinal cancer cells

**DOI:** 10.1039/d2ra90037h

**Published:** 2022-04-27

**Authors:** Laura Fisher

**Affiliations:** Royal Society of Chemistry Thomas Graham House, Science Park, Milton Road Cambridge CB4 0WF UK advances-rsc@rsc.org

## Abstract

Retraction of ‘Structural characterization of ginseng cyclopeptides and detection of capability to induce apoptosis in gastrointestinal cancer cells’ by Zhuo Liu *et al.*, *RSC Adv.*, 2019, **9**, 29847–29855, https://doi.org/10.1039/C9RA03965A.

The Royal Society of Chemistry hereby wholly retracts this *RSC Advances* article due to a significant amount of unattributed text overlap with articles by different author groups that were not cited, including articles published in *Scientific Reports* by Zhenjian Zhuo *et al.*^1^ and *RSC Advances* by Man Zheng *et al.*^2^

The authors were asked to comment and provide the raw data for this article but did not respond. The considerable amount of unattributed text overlap, and the lack of raw data, undermines the integrity and reliability of the entire article.

The authors were informed but have not responded to any correspondence regarding the retraction.

Signed: Laura Fisher, Executive Editor, *RSC Advances*

Date: 29th March 2022

## Supplementary Material
